# Mini Safe Havens for population recovery and reintroductions ‘beyond-the-fence’

**DOI:** 10.1007/s10531-022-02495-6

**Published:** 2022-11-12

**Authors:** Kiarrah J. Smith, Maldwyn J. Evans, Iain J. Gordon, Jennifer C. Pierson, Simon Stratford, Adrian D. Manning

**Affiliations:** 1 Fenner School of Environment and Society, The Australian National University, Acton, ACT 2601 Australia; 2 Department of Ecosystem Studies, Graduate School of Agricultural and Life Sciences, The University of Tokyo, Tokyo, Japan; 3 The James Hutton Institute, Dundee, DD2 5DA UK; 4 Central Queensland University, Townsville, QLD 4810 Australia; 5 Land and Water, CSIRO, Townsville, QLD 4810 Australia; 6 Lead, Protected Places Mission, National Environmental Science Program, Reef and Rainforest Research Centre, Cairns, QLD 4870 Australia; 7 Australian Wildlife Conservancy, Subiaco East, WA 6008 Australia; 8 Centre for Conservation Ecology and Genomics, Institute for Applied Ecology, University of Canberra, Canberra, ACT 2617 Australia; 9 ACT Parks and Conservation Service, Canberra, ACT Australia

**Keywords:** Predator-proof fence, Refuge, Soft-release, Spillover effect, Translocation tactics, Trophic rewilding

## Abstract

In response to the ongoing decline of fauna worldwide, there has been growing interest in the rewilding of whole ecosystems outside of fenced sanctuaries or offshore islands. This interest will inevitably result in attempts to restore species where eliminating threats from predators and competitors is extremely challenging or impossible, or reintroductions of predators that will increase predation risk for extant prey (i.e., coexistence conservation). We propose ‘Mini Safe Havens’ (MSHs) as a potential tool for managing these threats. Mini Safe Havens are refuges that are permanently permeable to the focal species; allowing the emigration of individuals while maintaining gene flow through the boundary. Crucial to the effectiveness of the approach is the ongoing maintenance and monitoring required to preserve a low-to-zero risk of key threats within the MSH; facilitating in-situ learning and adaptation by focal species to these threats, at a rate and intensity of exposure determined by the animals themselves. We trialled the MSH approach for a pilot reintroduction of the Australian native New Holland mouse (*Pseudomys novaehollandiae*), in the context of a trophic rewilding project to address potential naïveté to a reintroduced native mammalian predator. We found that mice released into a MSH maintained their weight and continued to use the release site beyond 17 months (525 days) post-release. In contrast, individuals in temporary soft-release enclosures tended to lose weight and became undetectable approximately 1-month post-release. We discuss the broad applicability of MSHs for population recovery and reintroductions ‘beyond-the-fence’ and recommend avenues for further refinement of the approach.

**Supplementary Information:**

The online version contains supplementary material available at 10.1007/s10531-022-02495-6.

## Introduction

Wildlife managers use fences to control the impacts of a range of threatening processes on declining fauna (Hayward and Kerley 2009). In reintroduction science, fences range from temporary soft-release enclosures (defined below) for single species reintroductions (de Milliano et al. 2016) to permanent fenced sanctuaries for the restoration of whole ecosystems (Legge et al. 2018; Kingsford et al. 2021). The effectiveness of these structures varies globally (Littlewood et al. 2020). Soft-release enclosures are typically small, inexpensive structures that contain individuals or small groups in-situ for an acclimation period determined by wildlife managers (Liu et al. 2016). In contrast, fenced sanctuaries are designed to exclude threats at the landscape scale to sustain whole populations of vulnerable species in-situ (Bombaci et al. 2018). Headstarting exclosures (Ross et al. 2021) may be considered an intermediary, since they can include large permanent fences (like the sanctuaries defined above), but are functionally transient to individuals (like soft-release enclosures). The primary aim of headstarting exclosures is to provide protection for the small or dependent life stage of a species, either in known breeding grounds (Young et al. 2013), or as in-situ breeding enclosures (Short and Turner 2000).

Soft-release and headstarting exclosures are not suited for situations where the focal species remains highly vulnerable to the threats present in the surrounding landscape (i.e., ‘beyond-the-fence’; Evans et al. 2021) in their adult life stage (Moseby et al. 2014). For example, a population of western barred bandicoots (*Perameles bougainville*), successfully reintroduced with the aid of a headstarting exclosure, was extirpated by the incursion of exotic predators (Short 2016). While fenced sanctuaries are a short- to medium-term solution for the conservation of such vulnerable species, the ultimate goal is to have them persisting beyond-the-fence, amongst the threats that are challenging or impossible to eliminate (Hayward and Kerley 2009; Legge et al. 2018; Evans et al. 2021, 2022). To this end, researchers have been trialling a range of tactics to overcome inappropriate anti-predator responses (i.e., naïveté; Ross et al. 2019), support the retention or learning of beneficial behaviours, and harness the adaptive potential of species (Olla et al. 1998; Griffin et al. 2000; Shier and Owings 2007; Blumstein et al. 2019; Tetzlaff et al. 2019; Evans et al. 2021). The time required for behavioural and evolutionary changes to occur depends on many factors, including the phenotypic plasticity of the focal species (Price et al. 2003), and whether individuals have experience with the threat (or an analogue) in their lifetime or evolutionary history (Griffin et al. 2000; Jolly et al. 2021).

A tactic that could benefit the persistence of naïve native species where soft-release or headstarting exclosures may not be enough, is the ‘Small Mammal Refuges’ (SMRs) proposed by Smith and Quin (1996), which can provide unlimited time with guarded exposure to novel threats. Originally conceived in 1996 in the context of Australian small mammal conservation, the basic concept of SMRs is that of a predator-proof fence large enough to accommodate a core breeding area (similar to headstarting) that is permanently permeable to the focal species (i.e., not requiring assisted translocation, Smith and Quin 1996; Ross et al. 2021). The permeability, the authors proposed, allowed for the emigration of dispersing individuals into the surrounding area (creating a halo, or spillover effect) (Agarwal and Bode 2019; Moseby et al. 2020). Although emigration from fenced sanctuaries (Short and Hide 2015; Recio et al. 2016) and marine protected areas (Di Lorenzo et al. 2020) has been recorded, to our knowledge, only one study has empirically tested the SMR tactic (Short et al. 2017). That study’s 17 ha SMR for the greater-stick nest rat (*Leporillus conditor*) ultimately proved unsuccessful because it did not exclude predation by native sand monitors (*Varanus gouldi*), which were a key threat the rats were naïve to (Short et al. 2017).

Wildlife managers will be increasingly confronted with the challenge of managing the impacts of exotic *and* native predators as interest grows in trophic rewilding (i.e., multi-trophic species restoration, Svenning et al. 2016; Fernández et al. 2017; Sweeney et al. 2019; Gaynor et al. 2021). In a recent essay, Evans et al. (2022) identified SMRs as a tactic under the explicit theme of coexistence conservation, which was defined as “the long-term, iterative, and adaptive process to enable the coexistence of threatened species and invasive predators”. Here, we develop and refine the concept of SMRs, which we refer to as ‘Mini Safe Havens’ (MSHs); acknowledging their potential applicability to non-mammalian species and referencing the link to the term ‘safe havens’ often used to refer to large, fenced sanctuaries (Department of the Environment 2015; Ringma et al. 2018). Mini Safe Havens are refuges that are permanently permeable to the focal species; allowing for in-situ learning and adaptation to the key threats that led to the species’ extirpation or decline at a rate and intensity of exposure determined by the animals. Our study also constitutes a step towards the overarching goal of establishing the New Holland mouse (*Pseudomys novaehollandiae*, ‘New Hollands’), an Australian native rodent, throughout a landscape where it was potentially naïve to a previously reintroduced native predator. We asked whether, in the context of trophic rewilding, a MSH is more successful for supporting the persistence of the New Holland mouse than a more traditional soft-release approach? We expected mice to use the MSH for longer than soft-release enclosures, and that there would be emigration from the MSH. Based on our results, we outline the next steps in the refinement of MSHs to enable broader application of the approach in wildlife restoration and recovery beyond-the-fence.

## Methods

### Focal species

The New Holland mouse currently occurs primarily in coastal areas in south-east Australia. It is a nocturnal burrowing species and an opportunistic omnivore (Pye 1991; Wilson and Bradtke 1999), known to be capable of producing multiple litters in a year (believed to be dependent on food availability) with an average of four pups per litter (Kemper 1980; Fox et al. 1993). It is listed as vulnerable on the IUCN Red List, with a fragmented and decreasing population (Woinarski and Burbidge 2016). Previous reintroduction attempts for the New Holland mouse are limited to trials of soft-release enclosures and monitoring methods in the eastern Otways, Victoria (Wilson et al. 2003, 2017), and a soft-release to Mulligans Flat Woodland Sanctuary (MFWS, or ‘Sanctuary’) in 2013 (Abicair et al. 2020). Notably, the latter took place in the absence of any mammalian predators (i.e., a key threat to naïve New Hollands), with the population observed to be persisting when surveyed three years after release (Abicair et al. 2020). For the trials in the present study, we sourced New Hollands from a captive breeding colony at The Australian National University (Online Resource 1).

### Recipient site

**Fig. 1:**
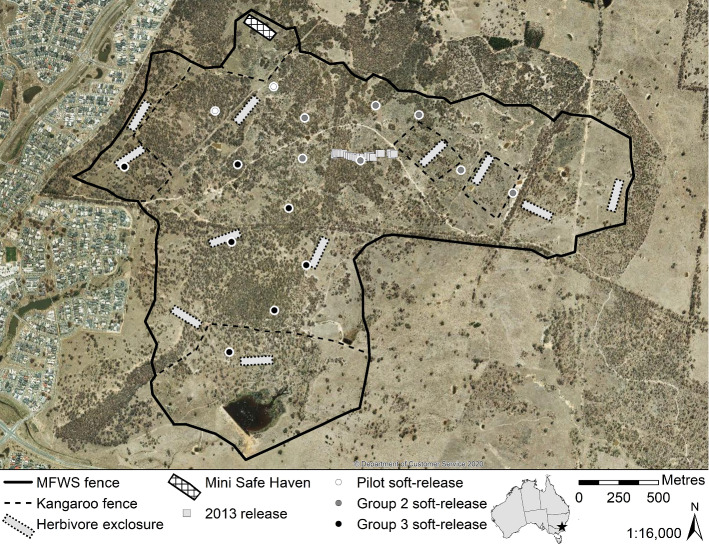
Mini Safe Haven and 16 soft-release sites (in three groups) in Mulligans Flat Woodland Sanctuary (MFWS). Experimental kangaroo exclusion fences impede, but do exclude, large macropods (Manning et al. 2011; Shorthouse et al. 2012). Similarly, herbivore exclosures function as an obstacle, rather than a barrier, to any species capable of jumping, climbing, digging under, or passing through, the fence. Location of the 2013 release sites (near the centre of MFWS) retrieved from Abicair et al. (2020)

Mulligans Flat Woodland Sanctuary encompasses approximately 485 ha of former agricultural land (Fig. 1) on Ngunnawal Country in the Australian Capital Territory (ACT, -35.1642, 149.1668) (Shorthouse et al. 2012). Ngunnawal names have been listed alongside species names, where known. The indigenous Ngarigo name ‘pookila’ was recommended for the New Holland mouse (Braithwaite et al. 1995), but has not been used here, because to date, it has not been adopted by the Ngunnawal community. The Sanctuary is the location of a long-term ecological experiment and ecosystem restoration project (Manning et al. 2011; Shorthouse et al. 2012) for the critically endangered “Box-Gum Grassy Woodlands and Derived Grasslands” community (McIntyre et al. 2010). A fence that permanently excludes exotic predators and herbivores, and livestock, was built in 2009 (Shorthouse et al. 2012). Reintroduced populations of eastern bettong (*Bettongia gaimardi*, ‘ngaluda’), New Holland mouse, eastern quoll (*Dasyurus viverrinus*, ‘murunguny’) and bush stone-curlew (*Burhinus grallarius*, ‘warabin’ and ‘mulyara’) were subsequently established (Batson et al. 2016; Manning et al. 2019; Abicair et al. 2020; Rapley 2020; Wilson et al. 2020, 2021) as part of the goal to rebuild trophic links (MFWS 2020).

We considered there was potential that a synergy of increased predation pressure following the reintroduction of eastern quolls in 2016, and reduced food and shelter due to drought (2017–2019 inclusive) (Lunney and Matthews 2004; BOM 2022) and heavy grazing by large macropods, could have had impacts on the original reintroduced (Abicair et al. 2020) population of New Hollands in MFWS. Despite employing the same seed-splitting detection technique as surveys undertaken in 2016 (Abicair et al. 2020), we detected no New Hollands (or house mice, *Mus musculus*) in MFWS during pre-release surveys undertaken in 2019 (details in Online Resource 1). While acknowledging the difficulty of detecting small or uncommon species like the New Holland mouse (Refsnider et al. 2011; Burns et al. 2019), we considered additional reintroduction trials necessary to help establish and maintain the species in the more challenging trophic rewilding context posed by the reintroduction of the eastern quoll; a mammalian predator that historically coexisted with the New Holland mouse. Although the beyond-the-fence setting in this case was within a fenced sanctuary, it nonetheless exemplifies the scenario where soft-release or headstarting exclosures may not be enough because the focal species is vulnerable to the threats present in the landscape. We structured our study across two trials to test two distinct approaches: Trial 1—soft-release, and Trial 2—MSH.

### Trial 1: soft-release

**Fig. 2:**
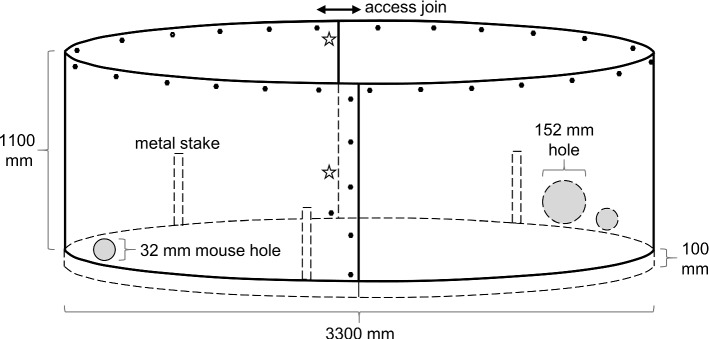
Ring-tanks were made from sheet metal (~ 1.2 mm thick), dug ~ 100 mm into the ground and internally supported by metal stakes secured to the walls. The ring-tank was accessible through a join held together by a bolt (stars) at the top and near the bottom (above ground level). All other joins were held together with screws (black dots). The two mouse holes and single large hole each had a removeable door (wider than the measurements given) made from 0.5 mm sheet aluminium held on with multiple bolts. Leafy branches and mouse houses were added to the ring-tanks for shelter. For Trial 2 only, we covered ring-tanks with fine fruit-netting held taut with fold-back clips. These clips were not strong enough to stop possums from jumping in. We suggest the use of screws (as illustrated) to hook the netting over the walls, instead of fold-back clips. All measurements are approximate. Dotted lines illustrate transparency. Illustration not to scale. See Online Resource 1 for additional construction notes and photos

The population of New Hollands released into MFWS in 2013, using a central, clumped soft-release approach (Fig. 1), had not expanded beyond 200 m after three years (Abicair et al. 2020). Given those findings, and the changed multi-trophic context, our next step towards the overarching goal of establishing New Hollands throughout MFWS was to refine the tactics for supporting population persistence. We used the Translocation Tactics Classification System (Batson et al. 2015), which has been proven to be highly effective in improving reintroduction success over successive trials (Wilson et al. 2020), to aid decision making, adaptively refine tactics and mitigate risks. Our approach for Trial 1 was to seed small groups over a broad area; encouraging the dispersal of individuals across the whole Sanctuary and alleviating the concentration of resource competition and predation risk in any one area (Kinnear et al. 2016; McGregor et al. 2020). We installed one ring-tank (Fig. 2) designed to temporarily exclude quolls, bettongs, and possums (*Trichosurus vulpecula*, ‘wilay’) at each of 16 sites across MFWS (Fig. 1). We staggered the releases of four male and four female New Hollands into each ring-tank in three groups (pilot, group 2, and group 3) between October-November 2019 to spread the monitoring and maintenance load (128 individuals total). We provided food and water daily (except when setting traps). After a holding period of 10–15 nights (varying between sites), the ring-tanks were made permeable to mice through two mouse doors, and the amount of food and water given in the ring-tanks was gradually reduced over a further 3–6 nights (varying between sites) to encourage dispersal across MFWS. After ceasing the supply of food and water in ring-tanks, we provided a daily scattering of sunflower seeds within ~ 10 m of the ring-tanks for a further 4–18 nights. Due to concerns that the risk of predation of New Hollands at the ring-tanks might be higher than the risk of mice not being able to find food for themselves, the latter two release groups in Trial 1 received a shorter period of sunflower seed scattering than did the pilot group. At the end of this temporary soft-release period, our daily visits ceased, and we made ring-tanks permeable to larger mammals (including quolls) to avoid these species becoming trapped within the ring-tanks. Additional tactics employed for Trial 1 are detailed in Online Resource 1.

### Trial 2: Mini Safe Haven

The tactics we employed for Trial 2 were adapted based on the post-release outcomes and opportunistic observations from Trial 1 (see *Results*). The overall aim of the MSH approach was to provide permanent safe habitat in which New Hollands could persist and disperse from. Working with the site fidelity observed in the soft-released captive-bred New Hollands (Trial 1), we created a permanent core area containing relatively abundant and diverse food and shelter, similar to the SMRs proposed by Smith and Quin (1996), protected from the risk of predation by quolls and heavy grazing by large macropods. We hypothesised that New Hollands could choose to stay or return to reproduce in this MSH, radiating out in their own time, rather than being held inside, or encountering an environment with a higher intensity of threats before they had settled in.

**Fig. 3:**
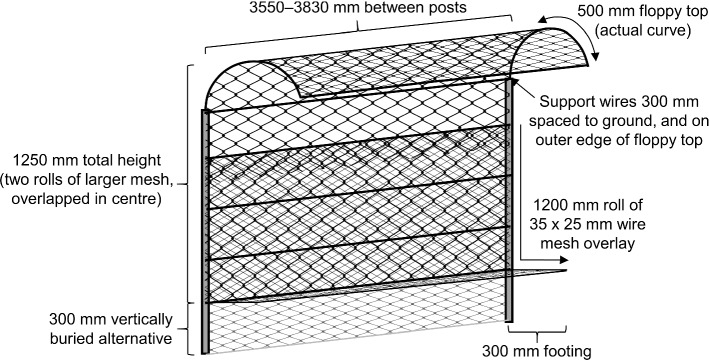
Our retrofitted Mini Safe Haven (MSH) fence with floppy top (to stop quolls climbing over; adapted from the design in Moseby and Read 2006), and 35 × 25 mm aperture wire mesh (permeable to mice but not quolls) secured over the pre-existing larger mesh. All measurements are approximate. The fence footing (preventing animals from digging underneath) was more easily pinned flush to the ground when grass was cut short before construction. We recommend constructing MSHs away from drainage lines and soggy soil, and suggest the footing be covered in gravel or buried vertically to reduce maintenance effort. Gravel may be more suitable where corrosive soils would damage the fence. Illustration not to scale. See Online Resource 1 for additional construction notes and photos

We retrofitted an existing herbivore exclosure fence (originally constructed to exclude bettongs, see Shorthouse et al. 2012) to permanently exclude quolls, large macropods, bettongs, and echidnas (*Tachyglossus aculeatus*, ‘burugun’) from an area of approximately 1 ha (~ 200 × 50 m) in MFWS (Figs. 1 and 3). The latter two species were not a threat to New Hollands, but could create pathways for quolls to enter the MSH by digging under the fence. The exclusion of intense grazing by large macropods for eight-years prior to retrofitting (Manning et al. 2011; Shorthouse et al. 2012; Evans et al. 2019) meant that our MSH contained relatively abundant tall and seeding grasses compared to the surrounding landscape; offering enhanced cover and food resources for New Hollands, compared to the Trial 1 sites located across the broader Sanctuary. We installed two ring-tanks (the same design as Trial 1) near the fence at the centre of our MSH. Unlike the Trial 1 ring-tanks, it was not necessary to make these permeable to larger mammals (to avoid inadvertently trapping them inside) because they were excluded by the MSH fence (Fig. 3). As a precautionary secondary barrier to quolls and avian predators, we covered the ring-tanks with taut, fine fruit-netting.

We translocated a total of 75 New Hollands into the MSH over a period of 10 days in March−April 2020 (it was necessary to undertake the translocation over multiple days due to limited person and vehicle availability). Each Trial 2 ring-tank received five male and five female New Hollands and was made permeable to mice after three nights. We released the remaining mice individually in wire-topped plastic laboratory rat tubs (i.e., in the same way they were kept in captivity) around the centre of the MSH. These mice were held for 1–2 nights before the tubs were opened and stick ladders added to allow mice to climb out, and back in, at their discretion. We checked the tubs daily (at the same time food and water was provided) and removed them as mice vacated. To anchor the New Hollands to the MSH, we scattered sunflower seeds throughout the site daily, gradually reducing this to three times weekly, and then once per week (ceasing at 12 months post-release). We did not supply supplementary water again until the Austral summer. Full details of our MSH design (including notes on construction) and release tactics are included in Online Resource 1.

### Post-release monitoring

Knowing that any predator incursion could threaten the persistence of a population (Frank et al. 2014; Short 2016), continuous maintenance and monitoring of the MSH was key to the approach. To do this, we used two tactics: (1) we installed a live-relay camera (Swift Enduro 4G) baited with sardines, peanut butter and oats that was continuously operational each night to detect any incursions, which would trigger a rapid response for removal; and (2) we undertook regular checks for gaps in the fence footing (which were pinned down when found) concurrently with supplementary feeding.

**Fig. 4:**
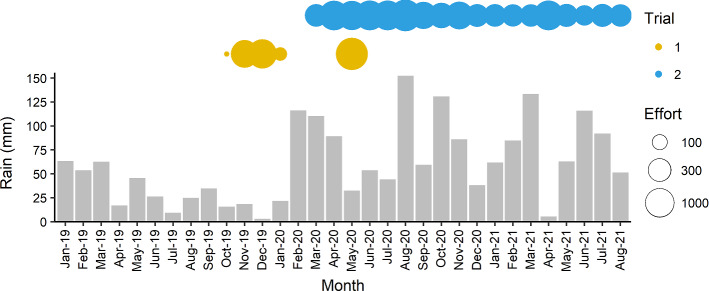
Monthly rainfall (BOM 2022) and total monthly survey effort (i.e., sum of ‘trap nights’ for all survey types) deployed for each trial. Trial 1 commenced during the final months of a three-year drought (Commonwealth of Australia 2020). Refer to Online Resource 1 for detailed survey effort and methods

We used Longworth traps (NHBS, Devon, UK) to capture mice in and around the Trial 1 and Trial 2 release sites, and where the presence of New Hollands was indicated by the seed-splitting detection technique (as developed by Abicair et al. 2020; hereafter, ‘seed surveys’), remote cameras, remote microchip scanners (Trial 2 only), or radio-tracking (Trial 1 only; fitted to 15 individuals using Holohil model BD-2NC, collars weighing 0.71–0.93 g). Due to logistical constraints (including Sanctuary closures and COVID-19 related restrictions) survey effort for New Hollands varied between sites and trials (Fig. 4). Refer to Online Resource 1 for details of survey methods.

### Data analyses

To determine the minimum number of New Hollands known to be alive at any one time, we collated records of individual presence and mortalities across all survey methods and opportunistic observations. For the Trial 1 remote camera data only, we distinguished individuals based on physical differences (e.g., ear notch or tail length). This was not possible for the Trial 2 camera data due to time constraints; hence a minimum of one New Holland mouse known alive could be deduced on any one night from cameras in Trial 2 (additional individuals were identified from live traps and microchip scanners). The same conservative estimate was applied to the results of sunflower seed surveys. Camera detections after midnight, but before sunrise, were recorded as the date of the previous day. Using the collated data, we computed Kaplan–Meier survival curves (Kaplan and Meier 1958; Rich et al. 2010). To test for a difference between trials we used the Peto-Peto test because most of the mortality events occurred at the beginning of the study, and there were many mice that became undetectable (i.e., censored, Etikan et al. 2018).

We used body mass as an indicator of health (McGuire et al. 2018), assuming those individuals that lost less weight post-release would be more likely to establish (Pearson et al. 2003). Fluctuations in weight of up to 10% are considered normal for New Hollands in captivity (A.D. Manning, unpublished data). Due to the innate stressors of the translocation procedure and wild environment, we anticipated that New Hollands could lose more than 10% of their body mass post-release. We tested for an effect of ‘trial’ and ‘time’ (days post-release) on ‘body mass’ by fitting a linear mixed model on a dataset containing one or more baseline measures of body mass (recorded between 0 and 37 days prior to release) for each individual, as well as the body mass of individuals recaptured post-release. The weight of implanted microchips is negligible and was not subtracted from body mass where present. We included ‘individual’ as a random factor to account for repeated measures.

Finally, we illustrated the frequency of New Holland mouse visits to baited cameras in Trial 2 over 162 nights between March and September 2020 on a hotspot map (Esri 2020) with annotations to show their dispersal over time. We only counted New Hollands that could be identified with certainty (i.e., not obscured by vegetation, fog, movement, etc.). All analyses and plotting were undertaken in R (R Core Team 2021; RStudio Team 2021). The main packages we used were ‘lmerTest’ (Kuznetsova et al. 2017), ‘AICcmodavg’ (Mazerolle 2020), ‘ggplot2’ (Wickham 2016), ‘survminer’ (Kassambara et al. 2021), and ‘survival’ (Therneau 2020).

## Results

### Detection

Assuming the site of detection was an individual’s release site, the longest time a New Holland mouse was detectable in Trial 1 was 36 nights post-release (with no captures or camera detections on 17 subsequently surveyed nights within the first 69 days post-release). Quoll incursion into Trial 1 ring-tanks occurred on three occasions (despite pre-release tests indicating the design excluded quolls). Additionally, after the mouse doors were opened, remote cameras recorded quolls capturing New Hollands at ring-tanks on four occasions.

Seed surveys approximately six months post-release detected no New Hollands at any of the Trial 1 sites. New Hollands were detected at nine stations in a comparable survey approximately five months after Trial 2 commenced, but not in either of the two subsequent Trial 2 seed surveys. In contrast to Trial 1, we were still detecting at least one New Holland mouse on cameras inside our MSH beyond 17 months (525 days) post-release, albeit at a declining frequency. Notably, house mice were not detected in any of our seed surveys but were present on remote cameras from late 2020 onwards, echoing increasing numbers of house mice in the region at the time (CSIRO and GRDC 2021).

**Fig. 5:**
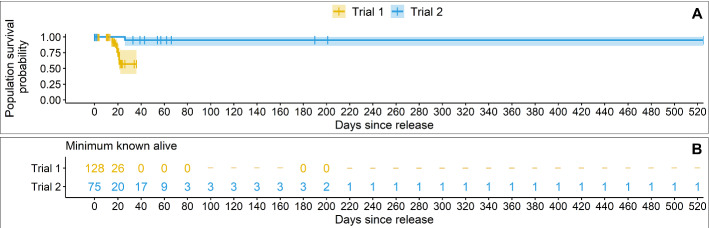
**A** Kaplan-Meier survival curves with confidence intervals based on **B** the minimum number of New Holland mice known alive post-release in each trial from live trapping, remote cameras, and microchip scanners. Censoring (i.e., last detection of individuals that we did not detect mortality for) is indicated in **A** by a vertical dash ‘|’. A horizontal dash ‘-’ in **B** indicates periods for which there was no survey effort. Cameras confirmed at least one individual was still present in the MSH at 525 days post-release. The survival probability should be interpreted as the probability of the population still being present

There was strong evidence for a difference between the trials in the probability of population persistence at any point in time (*χ*^*2*^(1) = 9.2, *P* = 0.002) (Fig. 5). Challenges with detectability (see “Discussion” section) meant that the fate of many individuals was censored (i.e., unknown). Given the Kaplan-Meier analysis assumes censored individuals will survive for as long as the last mouse known alive in their respective trial (Goel et al. 2010), the survival probability should be interpreted as the probability of the population still being present. No quoll or bettong incursions into the MSH occurred, but at least four echidnas dug underneath within the first year (prompting an immediate search and removal of the animal and repair of the fence footing). A single observed predation by a southern boobook owl (*Ninox novaeseelandiae*) was the only mortality confirmed in Trial 2.

### Body mass

In 2016, the mean body mass for wild New Hollands in MFWS was 13.6 g (Abicair et al. 2020). Of 124 individuals weighed post-release in Trial 1, ~ 17% maintained or gained weight (at one or more recaptures) and ~ 12% weighed less than 13.6 g. Of the lightest individuals, ~ 53% were in good condition or better (the body condition of two was not assessed). Of the records showing a loss of more than 20% body mass, half belonged to individuals that were overweight (> 25 g) or in excellent condition prior to release (i.e., they had more reserves to lose).

**Fig. 6:**
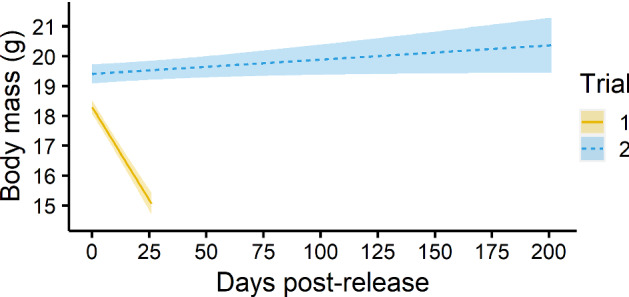
Change in New Holland mouse body mass over time post-release for each trial (predicted values with standard error)

Of 17 individuals weighed post-release in Trial 2, ~ 59% maintained or gained weight (at one or more recaptures) and the lightest was 15.5 g. No weight losses greater than 14% were recorded in Trial 2. Fewer individuals were recaptured in Trial 2 than in Trial 1 because no trapping took place during the period mice were held inside the ring-tanks (Online Resource 1). Overall, there was strong evidence that the body mass of individuals in Trial 2 was greater than that in Trial 1, and very strong evidence that the body mass of individuals significantly decreased over time. However, the change in body mass over time depended on trial (Table 1): Individuals’ weights declined in Trial 1 but were maintained in Trial 2 (Fig. 6). Although no females were pregnant prior to release, several post-release pregnancies were suspected in both trials for females with an increase in body mass of over 20%.

**Table 1:** Results of a linear mixed model that tested for an effect of ‘trial’ and ‘time’ (days post-release) on ‘body mass’ with ‘individual’ included as a random factor (variance 5.291 ± 2.3 SD)

Predictors	Estimate	Standard error	*P*	*F* (degrees of freedom)
Trial	1.12316	0.39278	0.00461**	8.1768 (1, 245.57)
Time	− 0.12426	0.01366	< 2e^− 16^***	68.7916 (1, 321.83)
Trial x time	0.12904	0.01440	< 2e^− 16^***	80.2573 (1, 321.83)

The strength of evidence for an effect is given by ‘*P*’, where ‘**’ indicates “strong evidence” and ‘***’ indicates “very strong evidence” (Muff et al. 2021). Degrees of freedom for the ‘*F*’ value were calculated using Satterthwaite’s method in an R “anova” (R Core Team 2021)

### Dispersal

**Fig. 7:**
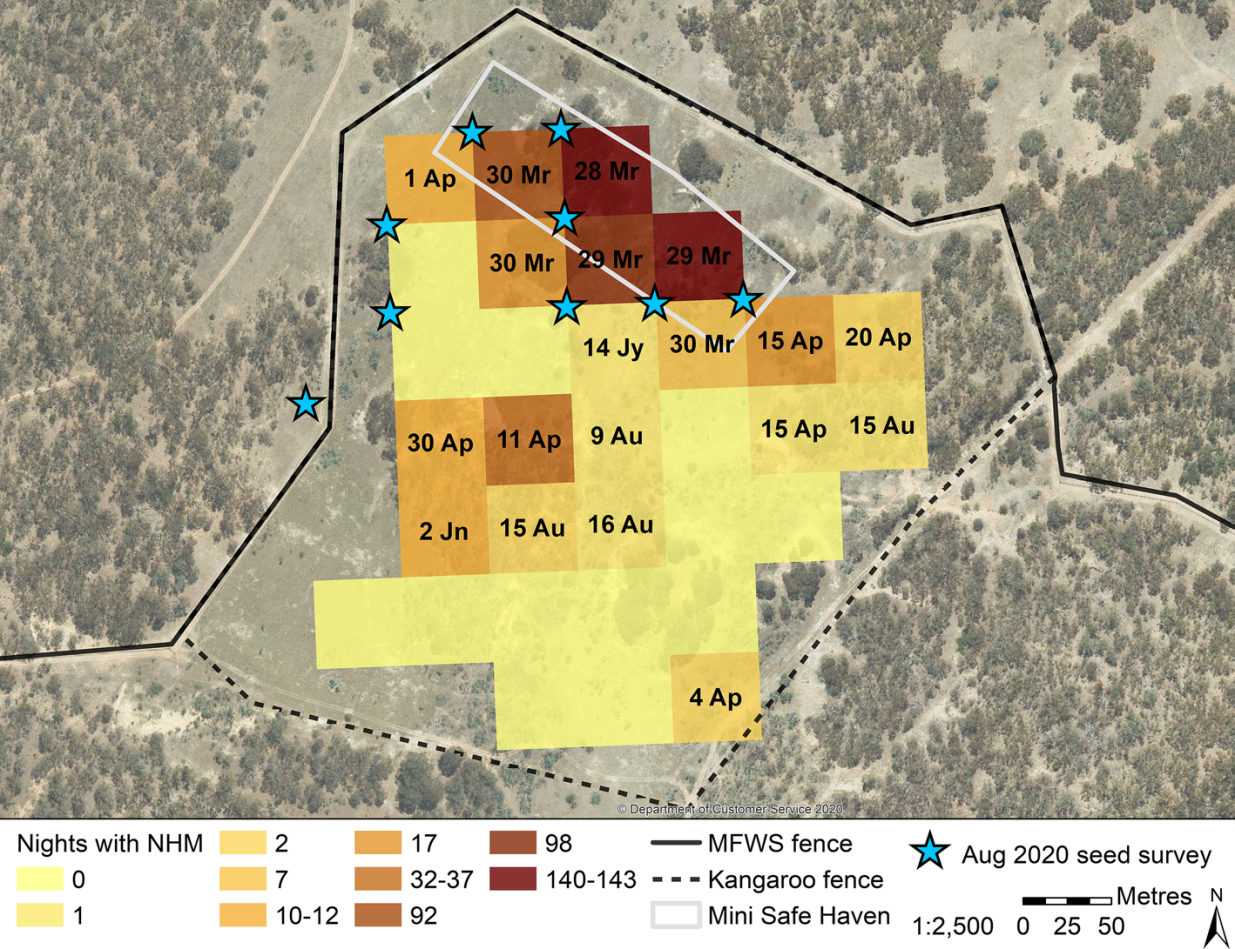
Frequency of New Holland mouse (NHM) detections on baited cameras (grid squares) in and around the Mini Safe Haven in Mulligans Flat Woodland Sanctuary (MFWS) over 162* nights between March and September 2020. Dates indicate the first night a New Holland mouse was detected at that location. Cameras without dates indicate locations where New Hollands were never detected. New Holland mouse detections in the August 2020 seed survey (stars) included one outside the western boundary of the MFWS fence. *The camera located in the north-west corner malfunctioned and was operational for 17 fewer nights

It was apparent that our monitoring approach for Trial 1 was not sufficient to detect individuals of this cryptic species dispersing away from the area immediately surrounding the ring-tanks. In Trial 2, both tank- and tub-released New Hollands were trapped or detected by microchip scanners in ring-tanks post-release. The baited camera array deployed until September 2020 detected individuals beyond ~ 200 m, though most activity occurred within that distance and inside the MSH (Fig. 7). Several camera sites outside the MSH were repeatedly visited by New Hollands, and over time, the number of sites visited by New Hollands increased (Fig. 7). Additionally, New Hollands were detected at one of the seed survey sites located outside the main MFWS fence (i.e., in the surrounding landscape without exclusion of exotic predators). Given that the New Hollands in Trial 1 became undetectable within approximately one month of release, and the dominant distribution of detections was close to the MSH, we assume that all detections around the MSH were from the Trial 2 release. However, we acknowledge it is possible that some detections may have been individuals released from neighbouring soft-release sites during Trial 1 (Fig. 1).

## Discussion

We found that a MSH was more effective than a traditional soft-release approach for supporting the persistence of a native rodent in an area that contains its native predators. New Holland mice released into a MSH maintained their weight and continued to use the release site beyond 17 months (525 days) post-release. In contrast, New Hollands in Trial 1, where we implemented a traditional soft-release approach, tended to lose weight and became undetectable at the release sites approximately one-month post-release (potentially dispersing beyond the area surveyed). Due to the Trial 1 ring-tanks’ small size, and shorter period of protection, we postulated the Trial 1 individuals probably had limited opportunity to acclimatise, become familiar with the natural food and shelter resources, or develop predator avoidance strategies. These effects were probably compounded by drought (Fig. 4), heavy grazing pressure, and potentially higher predation pressure because alternative prey may have been lacking in the environment. The rapid and unrecovered weight loss in Trial 1 may also be partly attributed to the stress of many individuals being held together in a small area (Olla et al. 1998; Long et al. 2005), and their more frequent recapture than in Trial 2, potentially producing trap-induced body mass declines (Pearson et al. 2003). Our results indicated that the MSH mitigated many of these issues, though we acknowledge a large amount of uncertainty owing to the inconsistent survey effort and limited number of New Hollands used, necessitated by the species’ threatened status, and amplified by the difficulties of detecting small or uncommon species (Refsnider et al. 2011; Burns et al. 2019).

Although we continued to see New Hollands on cameras in the MSH beyond the first seed survey for Trial 2, we suspect they were more difficult to detect in subsequent surveys because rainfall prompted a boom of food in the environment, potentially affecting the attractiveness of lures. This hypothesis is supported by the absence of house mouse detections, despite a regional mouse outbreak (CSIRO and GRDC 2021) and their presence on cameras from late 2020 onwards. The flourishing biomass in the latter half of Trial 2 contrasts with the environmental conditions of prevailing drought during the pre-release seed surveys, and in Trial 1 (Fig. 4). Since food for both prey and predator species was probably scarcer in 2019, the absence of New Holland mouse detections is likely to be indicative of an actual population decline between 2016 and 2019 (Abicair et al. 2020). Conversely, the lack of detections after the drought broke in 2020−2021 might instead reflect the limitations of seed surveys. Further study is required to clarify these limitations. Cameras were the most effective means of detecting New Holland mice in our study.

Our camera array recorded emigration from the MSH, with New Hollands expanding to new locations gradually over time. Similar to Abicair et al. (2020), we found most New Hollands within 200 m of the release site, though our monitoring beyond this distance was limited to seed surveys. The decline in frequency of New Hollands on cameras in the MSH suggests the presence of a source-sink gradient into the surrounding landscape (Moseby et al. 2020). Whether the sink represents dispersals or mortality is unknown. However, the repeated observations of New Hollands on cameras outside the MSH suggests that at least some individuals learned to persist in the presence of quolls, which at the time, had an estimated total population in MFWS of ~ 36 individuals (B.A. Wilson, personal communication 11th October 2021). This contrasts with the observed quoll predations in Trial 1. The capacity for naïve captive-bred New Hollands to learn to coexist with quolls, given time to learn and the security of the MSH in the landscape, may reflect a history of evolution in sympatry (Jolly et al. 2021). The MSH effectively created a refuge for the New Holland mouse (Pavey et al. 2017), with relatively tall and structurally complex ground vegetation, and no mammalian predators or large competitors. The MSH approach, therefore, potentially offers some opportunity to mitigate some of the major barriers to re-establishing the species’ adaptation to the climate, floristics, soils, and interspecific interactions of the formerly occupied inland grassy woodland habitat (Ford 2003; Canale and Henry 2010; Catullo et al. 2015). Below we discuss the broad applicability of MSHs for protecting extant populations and reintroducing and recovering species beyond-the-fence and recommend avenues for further refinement of the approach.

### Advantages of MSHs

The MSH approach is one of several innovations exploring the capacity for native prey to coexist with both native and exotic predators by manipulating ecology of fear dynamics (Blumstein et al. 2019; Tetzlaff et al. 2019; Evans et al. 2021, 2022; Gaynor et al. 2021; Manning et al. 2021). Mini Safe Havens alter both the actual risk of predation and individuals’ perception thereof (i.e., the ‘landscape of fear’, Laundré et al. 2010; Pentland 2014; Gaynor et al. 2021), allowing unlimited time for in-situ learning and adaptation to key threats beyond-the-fence. By contrast, a traditional soft-release provides only limited time for behavioural and evolutionary changes to occur in relative safety. Mini Safe Havens, in common with the SMRs, have two important advantages. First, when compared to large, fenced sanctuaries, they have relatively low costs for construction and maintenance (Smith and Quin 1996). Second, they allow the focal species to use the surrounding landscape and mix within the metapopulation, while maintaining a safe area for individuals to find refuge and reproduce (Smith and Quin 1996). Additionally, as we demonstrated here, MSHs aid the long-term monitoring of a species that is difficult to detect because, unlike temporary soft-release structures, they encompass locations where we expect a subset of the population to be consistently present.

The coexistence of competitors, predators and prey facilitated by MSHs could allow multi-trophic species restoration to proceed more rapidly than would be possible by reintroducing species in a predetermined sequence. By protecting known (or creating new) refuges, MSHs also have the potential to help recover and sustain extant but vulnerable populations through periods of drought (Pavey et al. 2017) (including via the provision of water within the MSH), and hyperpredation (where an abundance of non-native prey leads to an increase in predation pressure on the focal species, Smith and Quin 1996; Short et al. 2017). Mini Safe Havens may also support the rapid regeneration of vegetation and recolonization by individuals after fire (Kutt and Woinarski 2007; Legge et al. 2019) reducing the impact of predators that take advantage of burnt open habitats (Leahy et al. 2016). They can provide a physical buffer around soft-release sites and artificial refuges to eliminate visitation by competitors and predators and improve utilisation and reproductive outcomes for the focal species (Kemp et al. 2015; Keiter and Ruzicka 2020; Cowan et al. 2021; Chock et al. 2022). They could also enhance extant populations of uncommon species, allowing for the sustainable collection of more animals from the wild (as a more cost-effective alternative to captive breeding), increasing the likelihood of reintroduction success (Fischer and Lindenmayer 2000).

**Fig. 8:**
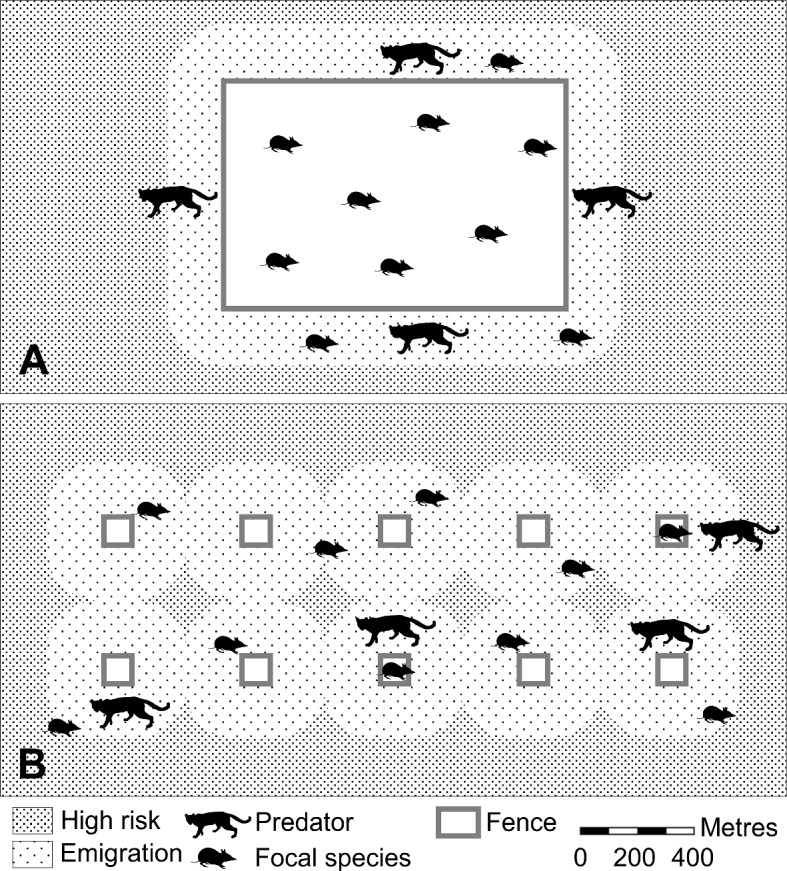
For a given length of fencing, the total area beyond-the-fence that is within the dispersal ability of a focal species is smaller for a fenced sanctuary (**A**) than for multiple Mini Safe Havens (**B**) in a landscape. A Mini Safe Haven network is likely to disperse predation risk for the focal species while minimally impeding the migration of other species. Silhouettes from phylopic.org, Public Domain Dedication 1.0

The use of multiple MSHs in a landscape where key threats persist may further aid reintroduction and recovery efforts by: increasing the amount of optimal habitat patches (Gardiner et al. 2018); dispersing a given number of predators over a larger area (McGregor et al. 2020, Fig. 8); reducing the risk of losing an entire population in the event of predator incursion (Smith and Quin 1996); and seeding a metapopulation to establish a species in a shorter time frame, over a larger area, varying terrain, and/or multiple land holdings (Lunney and Matthews 2004; Bode et al. 2012; Dickman 2012), while minimally impeding the migration of other species (Hayward and Kerley 2009). Regarding the spacing of multiple MSHs, it is interesting to note that emigration from marine protected areas generally occurs within 200 m and does not exceed 1 km (Di Lorenzo et al. 2020), which is similar to the ~ 200–250 m movements observed for several Australian rodents, including the New Holland mouse (Short et al. 2017; Abicair et al. 2020; Moseby et al. 2020). Additionally, it is logical that with increasing distance, individuals would be less likely to detect neighbouring MSHs, or encounter them by chance, while being more likely to encounter misadventure. More research is needed on optimal distances between adjacent MSHs. At a minimum, we expect that managers would seek to match the cost-per unit-area likely to be used by a metapopulation over a cluster of MSHs, with that of a single landscape-scale fenced sanctuary, requiring a simple calculation for the spacing between MSHs in a grid-layout (Fig. 8). Unlike a set of impermeable enclosures (Helmstedt et al. 2014), the areas between MSHs can be counted in this calculation (Dickman 2012), provided they are within a distance reasonable for the dispersal ability of the focal species (Fig. 8).

### Refining MSHs

In the same way that enforcement of marine protected areas is important to reap the benefits for marine conservation and fisheries (Guidetti et al. 2008), we found that maintenance and monitoring of the MSH was key; MSHs are not a ‘set-and-forget’ approach. Within the scope of the MSH approach there is opportunity to further refine the component tactics through future trials (Kemp et al. 2015). Size and design will depend on the focal species and the threats present beyond-the-fence in a given landscape. For example, the fence design experiments by Moseby and Read (2006) suggest that the MSH design used in our study may exclude cats and foxes, though the results of Robley et al. (2007) suggest a greater fence height might be necessary. Tests of MSHs are required to establish their effectiveness for excluding these species. Further modifications are likely to be necessary if the primary threat to the focal species is an avian predator; in this case, the addition of wire apparatus (Curtis et al. 1996), or mesh shelters (Arthur et al. 2005) may be trialled.

With refinement, MSHs would be suitable for the reintroduction of a range of species—not just mammals—(including multi-species releases) and could be scaled up for medium-sized species with the use of microchip-automated doors (Edwards et al. 2020; Watson et al. 2021), or species-specific sensors (Read et al. 2019) that exclude similarly sized predators and competitors. Although the MSH approach is best suited to species that have naturally low dispersal capacity, or display site fidelity, the latter may be artificially created, for example, by selecting for a particular phenotype (Fraser et al. 2001; Wilson et al. 2022); mating females at or just before release (Wilson et al. 2020); or experimenting with acclimation tactics, including supplementary feeding, as we did in our trials (Bright and Morris 1994; Knox et al. 2017; Jensen et al. 2021).

Smith and Quin (1996) recommended that SMRs be large enough to encompass the home ranges of a moderate to large number (> 10) of breeding females. However, one should remember that with increasing size, predator and competitor incursions will become harder to detect and manage (Smith and Quin 1996; Short et al. 2017). In determining a species-appropriate size for MSHs, we recommend instead that they be large enough to prevent the immediate dispersal of all animals at release, contain sufficient natural resources representative of the surrounding landscape for the focal species to interact with, and protect several females’ nesting sites (not their entire home ranges). For example, species similar to bank voles (*Myodes glareolus*) in high quality habitat might be suited to a network of MSHs of just 0.25 ha in size (Koskela et al. 1999), whereas optimal breeding by 10 female pygmy rabbits (*Brachylagus idahoensis*) could be sustained by a MSH of 1 ha (based on the density obtained in breeding enclosures, DeMay et al. 2016). Ultimately, decisions on the MSH size and number should be made based on the size and number of the species proposed for reintroduction, and the species’ mobility, site fidelity, and potential for territoriality and aggressive interactions (Lovari et al. 2020). Practitioners should also be cognisant of habitat quality, the risk of density-dependent disease (Warren et al. 2003), and the intensity and type of threats beyond-the fence.

### Synthesis and applications

Establishing multiple populations of species across their previous distribution, within the context of multi-trophic species restoration, offers challenging conditions for the conservation of subordinate competitors (Chock et al. 2022) and species situated lower in the trophic web. However, with the growing interest in trophic rewilding in landscapes beyond-the-fence where key threats persist (Svenning et al. 2016; Fernández et al. 2017; Sweeney et al. 2019), wildlife managers throughout the world will increasingly face this challenge (Evans et al. 2022). Here we have shown, using a native Australian rodent, how tactics can be developed to manage interspecific interactions and support population persistence so that multi-trophic restoration can proceed. In addition to the reintroduction of prey and subordinate species, MSHs may also protect prey where a native predator is to be reintroduced, or aid in the recovery of extant but fragmented and declining populations of vulnerable species. We encourage further trials and experimentation with MSHs in a variety of environments to refine the approach and uncover its full potential as a complementary tool for wildlife conservation and pest impact-mitigation efforts.

## Supplementary Information

Below is the link to the electronic supplementary material.

Supplementary material 1 (PDF 1554 kb)

## Data Availability

The datasets analysed during the current study are available from the corresponding author on reasonable request.
