# Supplementary material for: Mini Safe Havens for population recovery and reintroductions ‘beyond-the-fence’
Source: Biodivers Conserv. 2022 Nov 12;32(1):203–25. doi: 10.1007/s10531-022-02495-6 (PMC9652606; doi:10.1007/s10531-022-02495-6)
Supplement: Supplementary file 1 — Supplementary material 1 (PDF 1554 kb) [file 10531_2022_2495_MOESM1_ESM.pdf]

Online Resource 1

Mini Safe Havens for population recovery and reintroductions 'beyond-the-fence'

Biodiversity and Conservation

Kiarrah J. Smith[1]*, Maldwyn J. Evans[1,2], Iain J. Gordon[1,3,4,5,6], Jennifer C. Pierson[1,7,8], Simon Stratford[9], Adrian D. Manning[1]

[1] Fenner School of Environment and Society, The Australian National University, Acton, ACT, 2601, Australia.

[2] Department of Ecosystem Studies, Graduate School of Agricultural and Life Sciences, The University of Tokyo, Tokyo, Japan.

[3] The James Hutton Institute, Dundee, DD2 5DA, UK.

[4] Central Queensland University, Townsville, QLD, 4810, Australia.

[5] Land and Water, CSIRO, Townsville, QLD, 4810, Australia.

[6] Lead, Protected Places Mission, National Environmental Science Program, Reef and Rainforest Research Centre, Cairns, QLD, 4870, Australia.

[7] Australian Wildlife Conservancy, Subiaco East, WA, 6008, Australia.

[8] Centre for Conservation Ecology and Genomics, Institute for Applied Ecology, University of Canberra, Canberra, ACT, 2617, Australia.

[9] ACT Parks and Conservation Service, Canberra, ACT, Australia.

*** Correspondence**

Kiarrah J. Smith: kiarrah.smith@anu.edu.au

Captive breeding

We collected a total of 23 New Hollands from wild populations on the north-coast of New South Wales (-32.2440, 152.5405; -32.1261, 152.4349) in 2016 and 2018. Ultimately, 16 of these New Hollands successfully contributed to a captive breeding program at The Australian National University, which was set up to provide a large number of individuals for reintroductions without negatively impacting the source populations.

Pre-release surveys

We used the seed-splitting detection technique (Abicair et al. 2020) to assess the occurrence of New Hollands in MFWS prior to our reintroduction trials. Seed surveys indicate presence based on between-species differences in how sunflower seeds are opened: New Hollands neatly split the seed hull, while house mice shred the hull, creating rough edges and chew marks (Abicair et al. 2020). We deployed seed stations between May and September 2019 in the area up to 1 km from the 2013 release site (*main text Fig. 1*), reflecting the maximum distance New Hollands have been recorded to travel (Lazenby et al. 2007). Our seed stations comprised Longworth traps (NHBS, Devon, UK) that were locked 'open' with 20 undamaged sunflower seeds inside. Seeds were microwaved in batches on high power for approximately two minutes to enhance their scent and prevent germination. 875 trap nights were focused on the locations where New Hollands were detected in 2016 (Abicair et al. 2020), where one trap night was equal to one seed station present for one night. For 956 trap nights in the broader area, a metal excluder was secured over the top of each station to prevent disturbance by bettongs and possums. A final 528 trap nights took place with excluders and the addition of vanilla essence to the seeds.

Additional Trial 1 tactics

Our 16 Trial 1 ring-tanks were spaced >250 m, but <1 km apart, and evenly distributed between eucalypt woodland and stands of *Acacia* spp. within a grassland matrix. We chose this distribution to avoid assumptions (Britnell et al. 2021) about which habitat would be optimal for New Hollands given their status as a potential refugee species (Kerley et al. 2012; Abicair et al. 2020), meaning knowledge of their ecology is largely limited to the coastal habitats the species has become restricted to (i.e., not directly transferable to inland grassy woodland habitats). All sites included grass tussocks for shelter and eucalypt trees or *Acacia* spp. for shade. The latter was provided in captivity for enrichment and familiarisation with a potential food source (Keith and Calaby 1968).

After excluding individuals that were accidentally inbred, known to have seizures or freezing episodes (potentially a natural anti-predator response; Edut and Eilam 2003), in poor body condition, or with other health issues, we applied the PMx software Auto Cull function with DynamicMK (Lacy et al. 2012) to iteratively select individuals for release so that the retained captive colony's overall gene diversity was optimised. Individuals older than two years were selected first (n = 30; six of which were wild-born founders) because they were retired (or nearing retirement) from the captive breeding rotation. The same software allowed us to release together individuals that would have the most beneficial Mate Suitability Index (MSI) score, though many "very detrimental" groupings were unavoidable (Traylor-Holzer 2011). We also deliberately mixed the age of individuals (0.5 to 3.9 years) in each group.

We chose the Austral spring timing for the Trial 1 release as food resources were expected to be abundant for both New Hollands and their predators. Supplementary food provided in the ring-tanks included legumes, mushrooms, and bird seed. Further intervention

post-release was limited to filling in the gaps between the metal stakes and ring-tank walls after a mouse became trapped, and filling gaps dug by mice underneath ring-tank walls.

Additional Trial 2 tactics

We again used PMx to iteratively select individuals for release so that the captive colony's overall gene diversity was optimised, with individuals older than two years selected first (*n* = 6). To boost release numbers and avoid the alternative of euthanasia (a drastic reduction in colony size was required due to the limits imposed on animal technicians by COVID-19 lockdown restrictions in 2020), accidentally inbred mice (*n* = 13), individuals known to have seizures/freezing episodes (*n* = 19), and a single individual with a metabolic disorder, were also released. If seizure or inbred mice had any detrimental traits, we expected they would be removed from the MFWS population by natural causes.

The final cohort released into the MSH comprised 48 females and 27 males aged 0.9 to 2.5 years, two of which were wild-born founders. No genetic or age criteria were applied in grouping individuals for release into the Trial 2 ring-tanks. Accidentally inbred siblings of opposite sex were released >50 m apart to minimise the chance of further inbreeding. Possums could not be excluded from the MSH and proceeded to disturb some of the release tubs (*Fig. 1*). To prevent possums from taking the mouse food, we did not remove the lids completely. We chose to undertake the Trial 2 release in Austral autumn to avoid another hot and dry summer, but the timing was expedited by the need to rapidly reduce the caring requirements for the captive colony due to COVID-19 restrictions. Supplementary food provided in the MSH included sunflower seeds and legumes. We placed inverted laboratory rat tubs at the exits of the Trial 2 ring-tanks to guide New Holland mice towards the centre of the site and away from the adjacent MSH fence. Additional mouse shelters were placed throughout the MSH; supplementary logs had already been added as part of the broader

restoration experiment (Manning et al. 2011; Shorthouse et al. 2012). Ongoing maintenance and monitoring were supported by park rangers and volunteers (see *Acknowledgements*), requiring a minimum of four hours work per week, and many more hours for construction and in the early post-release phase.

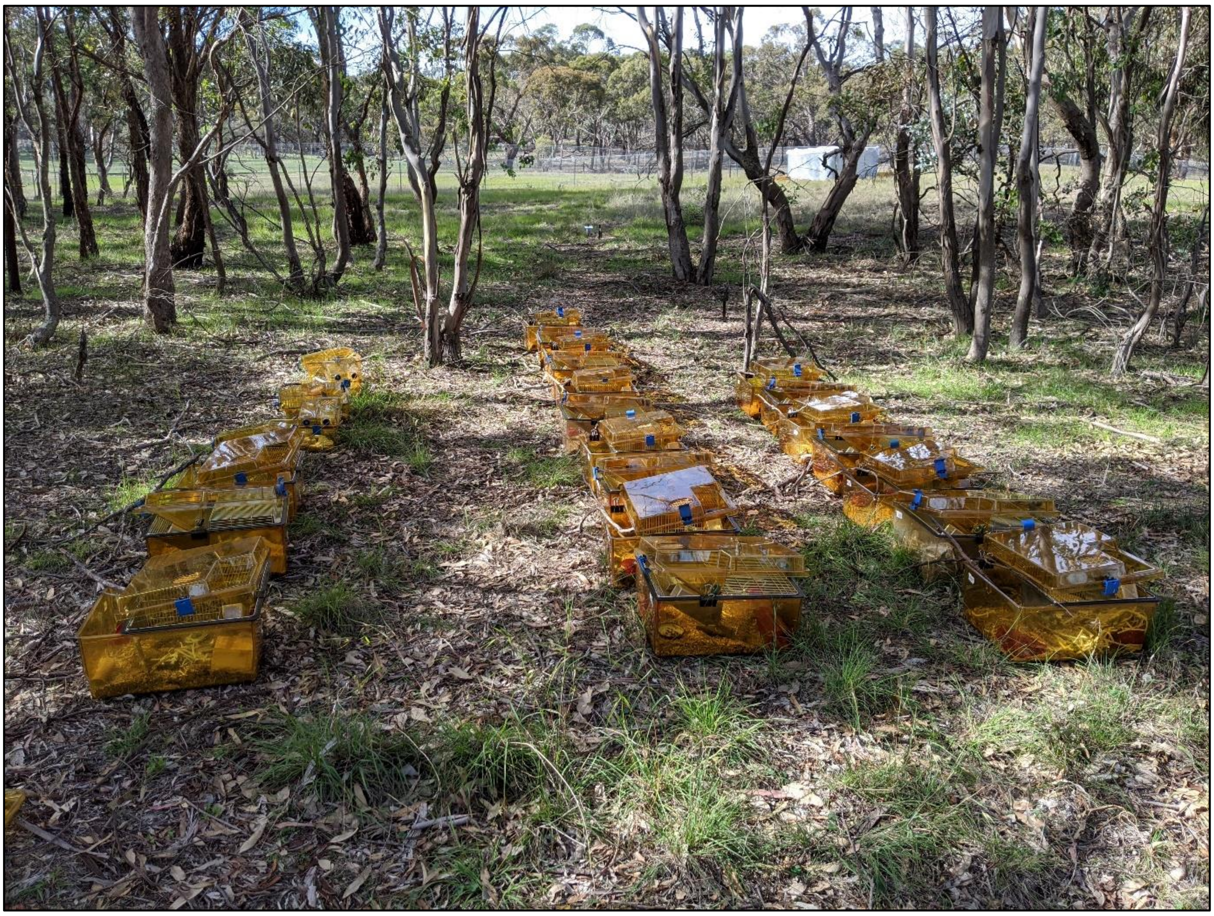

Fig. 1 A subset of the New Holland mice were released individually in wire-topped plastic laboratory rat tubs (i.e., in the same way they were kept in captivity) around the centre of the Mini Safe Haven

Construction notes

The ring-tanks (*Fig. 2*) were susceptible to coming out of the ground if: 1) buffeted by strong wind gusts and not buried deep enough or adequately supported by the internal stakes; 2) the gap between two sheets that could be opened to enter the ring-tank was held together at the base by a screw rather than a bolt; or 3) the walls were leant on. At least one mouse from two separate sites in Trial 1 left the ring-tank early by digging underneath the walls.

Trees overhanging the MSH fence (*Fig. 3*) were pruned or fitted with plastic trunk collars to prevent quolls climbing up and jumping over the fence. The frequency of pinning required for the fence footing was greatest in the first few months after retrofitting; we noted that rainfall could loosen the tent pegs holding it down, but that grass growing through the mesh helped to secure it. Fence maintenance should include the removal of tree saplings from the area of the fence footing and floppy top overhang. The floppy top was held up with heavy-gauge wire umbrella extensions available from Waratah Fencing (www.waratahfencing.com.au). Managers should be cognisant that thin gauge wire mesh may wear away faster than a thicker gauge.

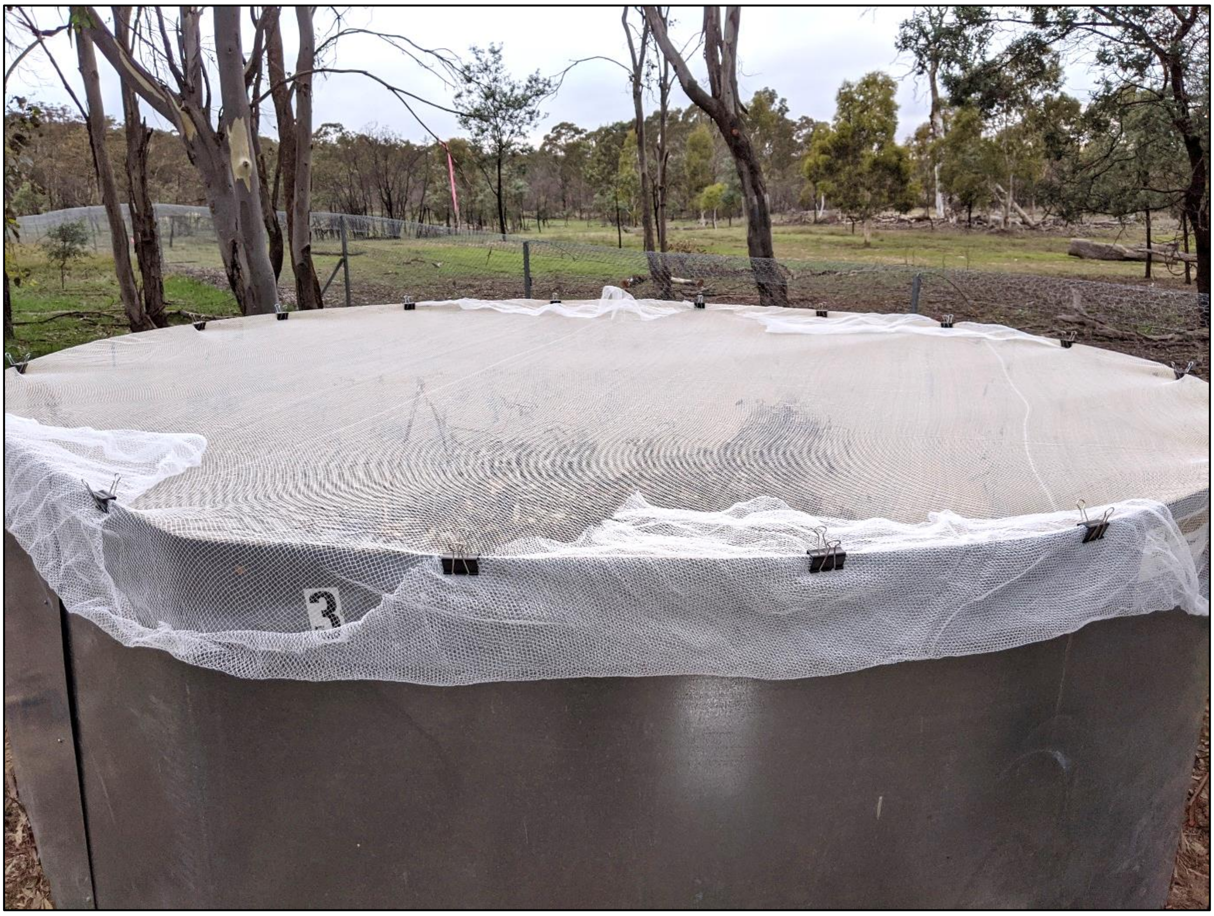

Fig. 2 Ring-tanks were made from sheet metal (~1.2 mm thick), dug ~100 mm into the ground and internally supported by metal stakes secured to the walls. The ring-tank was accessible through a join held together by a bolt at the top and bottom (above ground level). All other joins were held together with screws. Two mouse holes and single large hole each had a removeable door made from 0.5 mm sheet aluminium held on with multiple bolts. Leafy branches and mouse houses were added to the ring-tanks for shelter. For Trial 2 only, we covered ring-tanks with fine fruit-netting held taut with fold-back clips. These clips were not strong enough to stop possums from jumping in. We suggest the use of screws to hook the netting over the walls, instead of fold-back clips. Refer to the main text for an illustration with approximate measurements

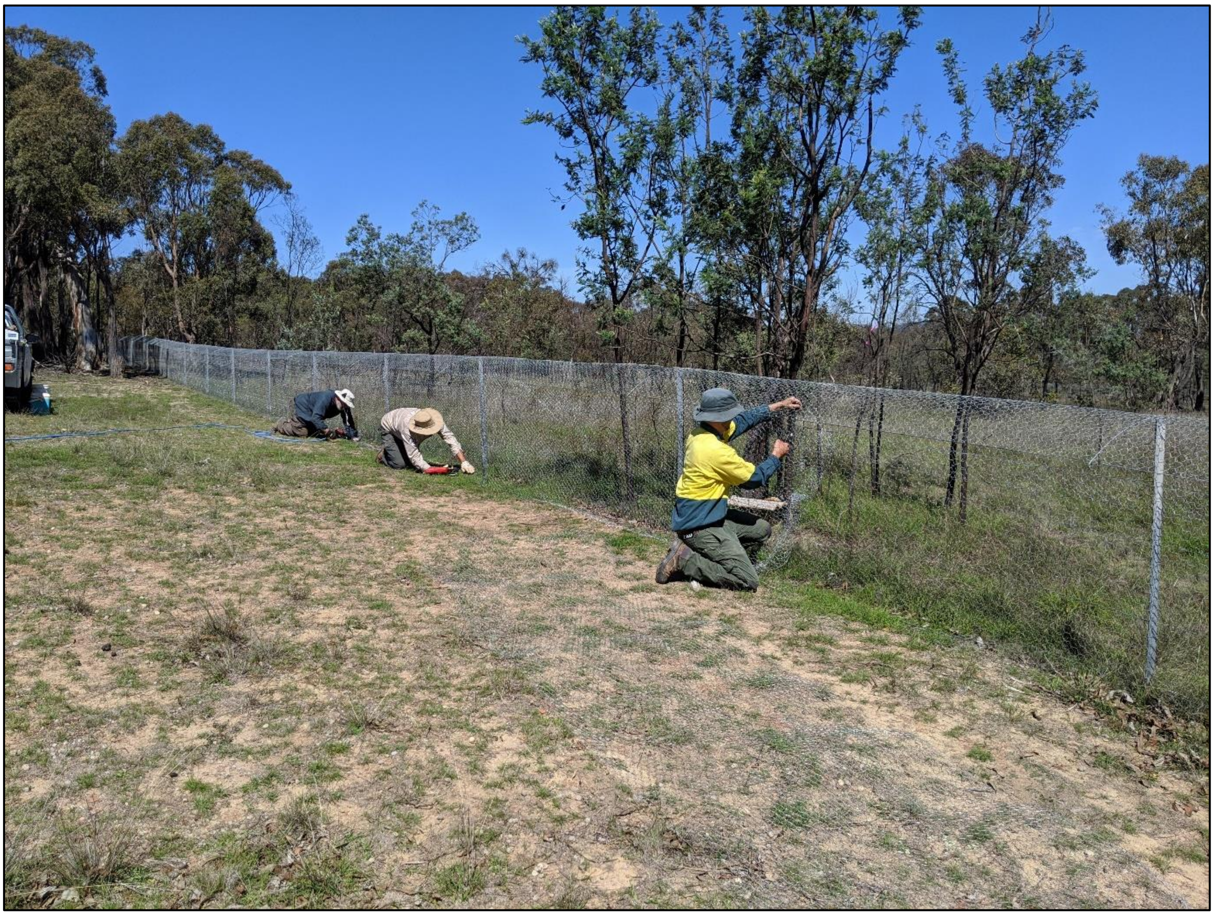

Fig. 3 Volunteers and Parks and Conservation Service Rangers constructing the retrofitted Mini Safe Haven (MSH) fence with floppy top and 35 x 25 mm aperture wire mesh (permeable to mice) secured over the pre-existing larger mesh. The fence footing was more easily pinned flush to the ground when grass was cut short before construction. We recommend constructing MSHs away from drainage lines and soggy soil, and suggest the footing be covered in gravel or buried vertically to reduce maintenance effort. Gravel may be more suitable where corrosive soils would damage the fence. Refer to the main text for an illustration with approximate measurements

Monitoring details

Our baited cameras were set to record at night and used a set up similar to that devised by Burns et al. (2017), with peanut butter, oats, vanilla essence, and golden syrup bait refreshed approximately weekly and allowed to be taken (in small amounts). In Trial 1, baited cameras were set at 64 sites across MFWS. For Trial 2, 28 baited cameras were deployed in a 50 m spaced grid south of the MSH (*main text Fig. 7*), with an additional four located within the MSH. In both trials, unbaited cameras were positioned to monitor mice entering and exiting ring-tanks after they were made permeable to mice. Additional unbaited cameras were moved between locations of interest (e.g., coarse woody debris, grass tussock tunnels, and burrows).

Longworth traps used for live trapping were baited with the supplementary feeding mix, a peanut butter oat ball, or sunflower seeds. Later trapping sessions included a piece of apple along with the peanut butter oat ball to provide trapped animals with a source of moisture. From each trapped individual, we recorded body mass, sex, body condition, and left small pes length (heel to the tallest foot pad, without the toes). Body condition was a subjective measure with four categories: poor, fair, good, or excellent (Ullman-Culleré and Foltz 1999). Mean small pes length was 16.51 mm (range 14.4–19.6 mm, n = 384).

In May 2020 (i.e., starting from between 180-211 nights post-release, varying between sites), all Trial 1 ring-tanks were surveyed with a 20 metre-spaced grid of 25 seed stations (without excluders) centred on the ring-tanks. The Trial 2 seed surveys at 144, 219 and 378 nights post-release comprised 145 seed stations (also without excluders) in a 50 metre-spaced array up to ~450 metres from the MSH. The microchip scanners used in Trial 2 were deployed around ring-tank exits and frequently visited camera baits. However, bettongs

chewed and damaged the antenna cables. *Table 1* summarises the monthly survey effort for each trial.

Table 1 Monthly effective survey effort (i.e., ‘trap nights’) for each trial. Numbers are approximate because some defective equipment was not accurately recorded

Month	Survey	Effective Trial 1	Effective Trial 2
Oct 2019	Live trapping	57	NA
	Unbaited ring-tank camera	2	NA
Nov 2019	Live trapping	459	NA
	Other unbaited camera	6	NA
	Unbaited ring-tank camera	201	NA
Dec 2019	Baited camera	544	NA
	Live trapping	349	NA
	Other unbaited camera	7	NA
	Unbaited ring-tank camera	53	NA
Jan 2020	Baited camera	83	NA
March 2020	Baited camera	NA	245
	Other unbaited camera	NA	5
	Unbaited ring-tank camera	NA	8
April 2020	Baited camera	NA	943
	Other unbaited camera	NA	28
	Unbaited ring-tank camera	NA	103
May 2020	Baited camera	NA	756
	Live trapping	NA	54
	Microchip scanner	NA	12

	Other unbaited camera	NA	29
	Seed survey	1995	NA
	Unbaited ring-tank camera	NA	124
June 2020	Baited camera	NA	960
	Live trapping	NA	18
	Microchip scanner	NA	6
	Other unbaited camera	NA	30
	Unbaited ring-tank camera	NA	120
July 2020	Baited camera	NA	992
	Microchip scanner	NA	1
	Other unbaited camera	NA	31
	Unbaited ring-tank camera	NA	124
August 2020	Baited camera	NA	992
	Other unbaited camera	NA	31
	Seed survey	NA	695
	Unbaited ring-tank camera	NA	119
September 2020	Baited camera	NA	477
	Other unbaited camera	NA	30
	Unbaited ring-tank camera	NA	120
October 2020	Baited camera	NA	124
	Live trapping	NA	177
	Other unbaited camera	NA	20
	Unbaited ring-tank camera	NA	124
November 2020	Baited camera	NA	120
	Other unbaited camera	NA	30

	Seed survey	NA	432
	Unbaited ring-tank camera	NA	120
December 2020	Baited camera	NA	113
	Other unbaited camera	NA	31
	Unbaited ring-tank camera	NA	124
January 2021	Baited camera	NA	103
	Other unbaited camera	NA	31
	Unbaited ring-tank camera	NA	124
February 2021	Baited camera	NA	105
	Other unbaited camera	NA	28
	Unbaited ring-tank camera	NA	112
March 2021	Baited camera	NA	96
	Other unbaited camera	NA	31
	Unbaited ring-tank camera	NA	124
April 2021	Baited camera	NA	120
	Live trapping	NA	118
	Other unbaited camera	NA	30
	Seed survey	NA	725
	Unbaited ring-tank camera	NA	120
May 2021	Baited camera	NA	124
	Other unbaited camera	NA	31
	Unbaited ring-tank camera	NA	124
June 2021	Baited camera	NA	119
	Other unbaited camera	NA	30
	Unbaited ring-tank camera	NA	30

July 2021	Baited camera	NA	124
	Other unbaited camera	NA	31
	Unbaited ring-tank camera	NA	124
August 2021	Baited camera	NA	60
	Other unbaited camera	NA	95
	Unbaited ring-tank camera	NA	97

References

Abicair K, Manning AD, Ford F, Newport J, Banks SC (2020) Habitat selection and genetic diversity of a reintroduced 'refugee species.' Anim Conserv 23:330-341. https://doi.org/10.1111/acv.12550

Britnell JA, Lewis RN, Elsner-Gearing F, Harvey N, Stanbrook E, Shultz S (2021) Species stereotypes as a result of unconscious research biases compromise conservation efficacy. Biol Conserv 261:109275. https://doi.org/10.1016/j.biocon.2021.109275

Burns PA, Parrott ML, Rowe KC, Phillips BL (2017) Identification of threatened rodent species using infrared and white-flash camera traps. Aust Mammal 40:188–197. https://doi.org/10.1071/am17016

Edut S, Eilam D (2003) Rodents in open space adjust their behavioral response to the different risk levels during barn-owl attack. BMC Ecol 3:1-16. https://doi.org/10.1186/1472-6785-3-10

Keith K, Calaby JH (1968) The New Holland mouse, *Pseudomys novaehollandiae* (Waterhouse), in the Port Stephens district, New South Wales. CSIRO Wildl Res 13:45-48. https://doi.org/10.1071/cwr9680045

Kerley GIH, Kowalczyk R, Cromsigt JPGM (2012) Conservation implications of the refugee species concept and the European bison: king of the forest or refugee in a marginal habitat? Ecography 35:519-529. https://doi.org/10.1111/j.1600-0587.2011.07146.x

Lacy RC, Ballou JD, Pollak JP (2012) PMx: software package for demographic and genetic analysis and management of pedigreed populations. Methods Ecol Evol 3:433-437. https://doi.org/10.1111/j.2041-210X.2011.00148.x

Lazenby BT, Pye T, Richardson A, Bryant SA (2007) Towards a habitat model for the New Holland Mouse *Pseudomys novaehollandiae* in Tasmania ? population vegetation associations and an investigation into individual habitat use. Aust Mammal 29:137-148. https://doi.org/10.1071/am07018

Manning AD, Wood J, Cunningham R, McIntyre S, Shorthouse D, Gordon I, Lindenmayer D (2011) Integrating research and restoration: the establishment of a long-term woodland experiment in south-eastern Australia. Aust Zool 35:633-648. https://doi.org/10.7882/az.2011.016

Shorthouse DJ, Iglesias D, Jeffress S, Lane S, Mills P, Woodbridge G, McIntyre S, Manning AD (2012) The 'making of' the Mulligans Flat – Goorooyarroo experimental restoration project. Ecol Manag Restor 13:112-125. https://doi.org/10.1111/j.1442-8903.2012.00654.x

Traylor-Holzer K (2011) PMx users manual, Version 1.0. IUCN SSC Conservation Breeding Specialist Group, Apple Valley, MN, USA.

Ullman-Culleré MH, Foltz CJ (1999) Body condition scoring: a rapid and accurate method for assessing health status in mice. Lab Anim Sci 49:319-323. https://pubmed.ncbi.nlm.nih.gov/10403450/
